# Use of bovine serum albumin might impair immunofluorescence signal in thick tissue samples

**DOI:** 10.1038/s41598-025-06876-z

**Published:** 2025-07-01

**Authors:** Anna Chwastowicz, Artur Wolny, Magdalena Sobień, Marcin Barański, Jacek Tomczuk, Michał Szatkowski, Aleksandra Szredzka, Jakub Gołąb, Leszek Kaczmarek, Marzena Stefaniuk, Paweł Matryba

**Affiliations:** 1 Department of Immunology, Medical University of Warsaw, 5 Nielubowicza Street, Warsaw, 02-097 Poland; 2 Laboratory of Neurobiology, BRAINCITY, Nencki Institute of Experimental Biology of Polish Academy of Sciences, 3 Pasteur Street, Warsaw, 02-093 Poland; 3 Laboratory of Imaging Tissue Structure and Function, Nencki Institute of Experimental Biology of Polish Academy of Sciences, 3 Pasteur Street, Warsaw, 02- 093 Poland

**Keywords:** Tissue clearing, Confocal microscopy, BSA, Bovine serum albumin, Immunofluorescence, Fluorescence imaging, Optical imaging, Immunohistochemistry, Confocal microscopy, Light-sheet microscopy

## Abstract

Significant progress in microscopic imaging techniques allowed transition from predominantly qualitative methods to a powerful tool for quantitative research, driven by improved instrumentation and computational power. Furthermore, previously limited to thin, laser-permeable tissue sections, imaging techniques have been revolutionized by the advent of tissue optical clearing. This innovation enables the visualization and quantitative analysis of entire organs and even whole bodies at cellular resolution. However, achieving high-quality imaging depends not only on the transparency of the tissue preparation but also on precise immunofluorescence labeling to ensure accurate signal detection and reliable study outcomes. In this study, we evaluated whether various reagents that are typically applied during the tissue blocking step prior to immunofluorescence staining affect the quality of the obtained image in thick and optically cleared samples. We demonstrate that the commonly employed tissue blocking step does not improve imaging conditions and even can substantially degrade fluorescence signal quality, particularly in large, optically cleared tissues such as whole mouse brain hemispheres.

**Supplementary Information:**

The online version contains supplementary material available at 10.1038/s41598-025-06876-z.

## Introduction

Over the past two decades, microscopy has evolved significantly, transitioning from primarily qualitative analysis to a robustly quantitative methodology, empowering researchers to explore intricate spatial relationships among cells through detailed visualization of their distribution. However, quantitative studies employing confocal microscopy have historically faced critical limitations in imaging depth. Tissue opacity often restricts imaging to superficial cell layers, with data quality deteriorating as imaging depth increases^1–3^.

Recent advances in tissue optical clearing (TOC) have revolutionized this field, overcoming depth limitations and enabling imaging of the entire laboratory rodent bodies at near-cellular resolution^4–6^. These breakthroughs have opened unprecedented opportunities to study complex scientific hypotheses within the three-dimensional context of whole organs, even in live animals^7,8^. However, the success of such studies requires meticulous attention at every stage of tissue preparation, from fixation and staining to imaging and data analysis. Each of these steps is crucial for generating unbiased results that form the foundation for subsequent experiments and high-stakes clinical studies.

Historically, autofluorescence and nonspecific antibody binding have been primary limitations in fluorescence imaging. To mitigate these issues, hundreds of protocols have been developed, including strategies to reduce autofluorescence and optimize antibody staining^9–11^. With the advent of TOC, a new challenge has emerged: achieving uniform antibody penetration in large tissue samples, often several centimeters in size. Proposed solutions include antibody perfusion^12^ chemical modifiers to improve tissue permeability^13–15^ and techniques like CUBIC-HistoVision^16^ which combines chemical additives (e.g., Quadrol and urea) with adjustments to temperature, ionic strength, detergent concentration, and limited tissue digestion.

Despite its transformative potential, TOC remains a time- and resource-intensive process. Preparing a sample, such as a mouse brain, can take one to two weeks using the most common protocols. Furthermore, successful imaging requires advanced instrumentation, such as light-sheet microscopy, and computational resources capable of handling datasets ranging from hundreds to thousands of gigabytes. Given these demands, optimizing each step of the workflow can substantially enhance research productivity.

Drawing inspiration from the observations of Buchwalow et al.^17^ who demonstrated that the blocking step in immunohistochemistry (originally intended to saturate Fc receptors and reduce nonspecific antibody binding) did not improve signal quality, we sought to investigate whether this finding extends to immunofluorescence. Specifically, we examined (1) whether omitting the blocking step affects signal quality in thin sections and (2) whether this step can be omitted in TOC without compromising image quality. Surprisingly, we found that skipping the blocking step not only does not degrade signal quality nor increase nonspecific binding, but in optically cleared tissues, it might enhance antibody penetration.

## Results

Buchwalow et al.^17^ using frozen and paraffin-embedded tissue sections, as well as cell culture monolayers and cytospins, demonstrated that the omission of a blocking step (with either 5–10% normal goat serum, NGS, or 1% bovine serum albumin, BSA in PBS) did not lead to the appearance of background or nonspecific antibody binding when using currently produced antibodies. To extend these findings, we sought to investigate whether these observations hold true in thicker, 50 μm sections of animal tissues in case of immunofluorescence. We selected lymph nodes and thymus for this analysis, as these lymphoid tissues are characterized by a high abundance of Fc receptors— presence of which has been described in the literature as responsible for nonspecific binding of Fc portion of antibodies.

**Fig. 1:**
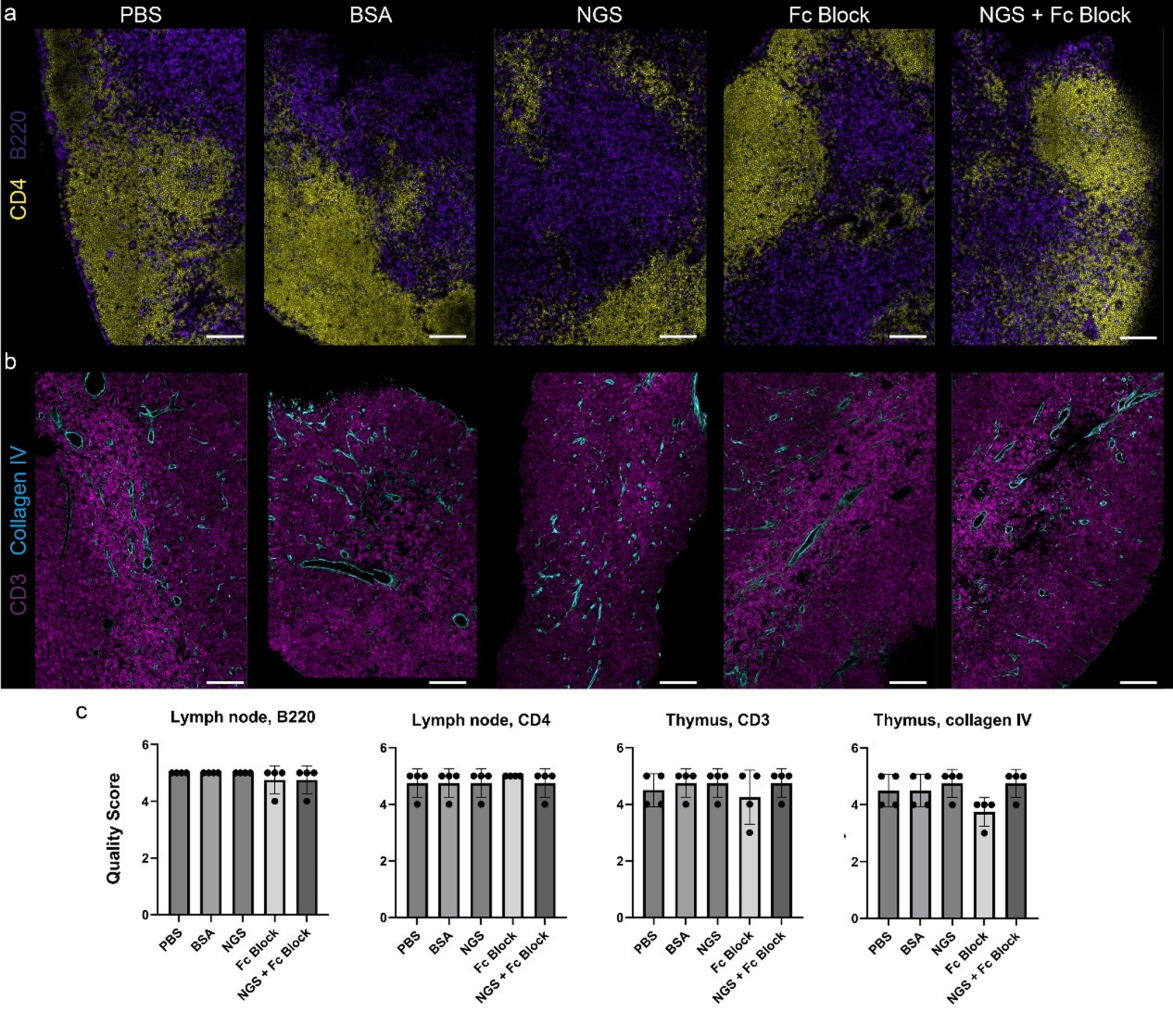
Immunofluorescence staining of murine lymphoid tissue samples processed with and without the protein blocking step prior to incubation with antibodies. (**a**) immunolabeling of murine lymph node against B220 (marker of B-lymphocytes) and CD4 (marker of T-helper lymphocytes) with secondary antibody conjugated with AF-488 and AF-647, respectively. (**b**) immunolabeling of murine thymus against collagen IV with secondary antibody conjugated with AF-568 and against CD3 (marker of T lymphocytes) with AF-647 directly conjugated antibody. (**c**) Four pathologists independently and blindly evaluated the quality of immunofluorescence staining in the panels (a) and (b), using a subjective scoring scale from 1 to 5, where 5 represents optimal conditions. No significant differences in image quality were observed between the PBS (no blocking agent) group and the other groups. BSA, bovine serum albumin, NGS, normal goat serum. Scale bar, 100 μm.

Regardless of the blocking solution used (BSA, NGS, Fc block, or a combination of NGS and Fc block), we observed no differences in image quality. This finding was consistent irrespective of using directly conjugated antibodies or a primary and secondary antibody staining protocol (Fig. 1 and Supplementary Figs. 1–2).

These initial observations were further confirmed by four pathologists, who were blinded to the experimental conditions and asked to rank the imaging files based on image quality and the subjective ratio of background to signal (Fig. 1c).

**Fig. 2:**
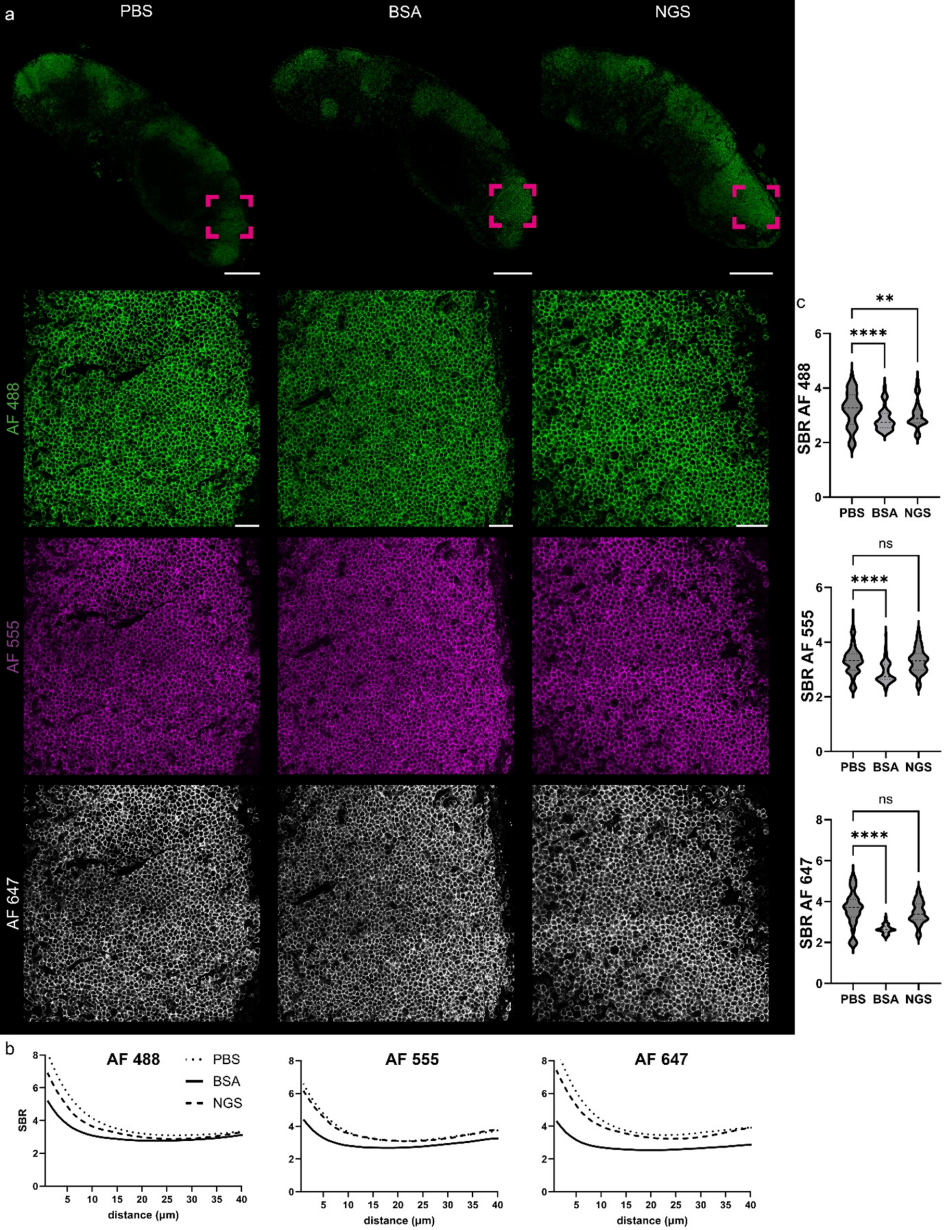
Protein blocking step with BSA diminishes signal-to-background ratio of commonly used fluorophores. (**a**) immunolabeling of murine lymph node against B220 (marker of B-lymphocytes) with secondary antibodies conjugated with AF-488, −555 and AF-647, ROI is zoomed in lower panels. (**b**) mean SBR values of three commonly used fluorophores across 40 μm imaging range, *n* = 7–8 per group and (**c**) SBR values pooled from 15 to 40 μm imaging range, one-way ANOVA with Dunn’s multiple comparison test, *n* = 7–8 per group; ****P < 0.0001, **P < 0.01. Scale bar, 300 μm and 30 μm in zoomed areas.

Encouraged by this qualitative findings, we aimed to quantify these observations for most commonly utilized blocking solutions and thus performed staining on the same lymph node sections, either blocked with BSA, blocked with NGS, or unblocked, and stained for the B220 marker, followed by a cocktail of three Alexa Fluor antibodies (AF-488, AF-555, and AF-647). Signal-to-background ratios (SBR) were plotted for each condition (Fig. 2). Interestingly, the SBR was notably lower in the BSA-blocked group for all fluorophores. To avoid the influence of high SBR values in the upper 15 μm of the sections—which could obscure significant differences in deeper parts of tissue—we conducted a focused analysis on the SBR between 15 and 40 μm deep into the tissue. This analysis confirmed that blocking with BSA resulted in a lower SBR compared to the unblocked group. Moreover, similar findings were observed when comparing the unblocked group to NGS blocking for AF-488, the wavelength spectrum where tissues exhibit higher autofluorescence compared to longer wavelengths (555/647). For AF-555 and AF-647, no statistically significant differences were detected between the unblocked and NGS-blocked conditions.

**Fig. 3:**
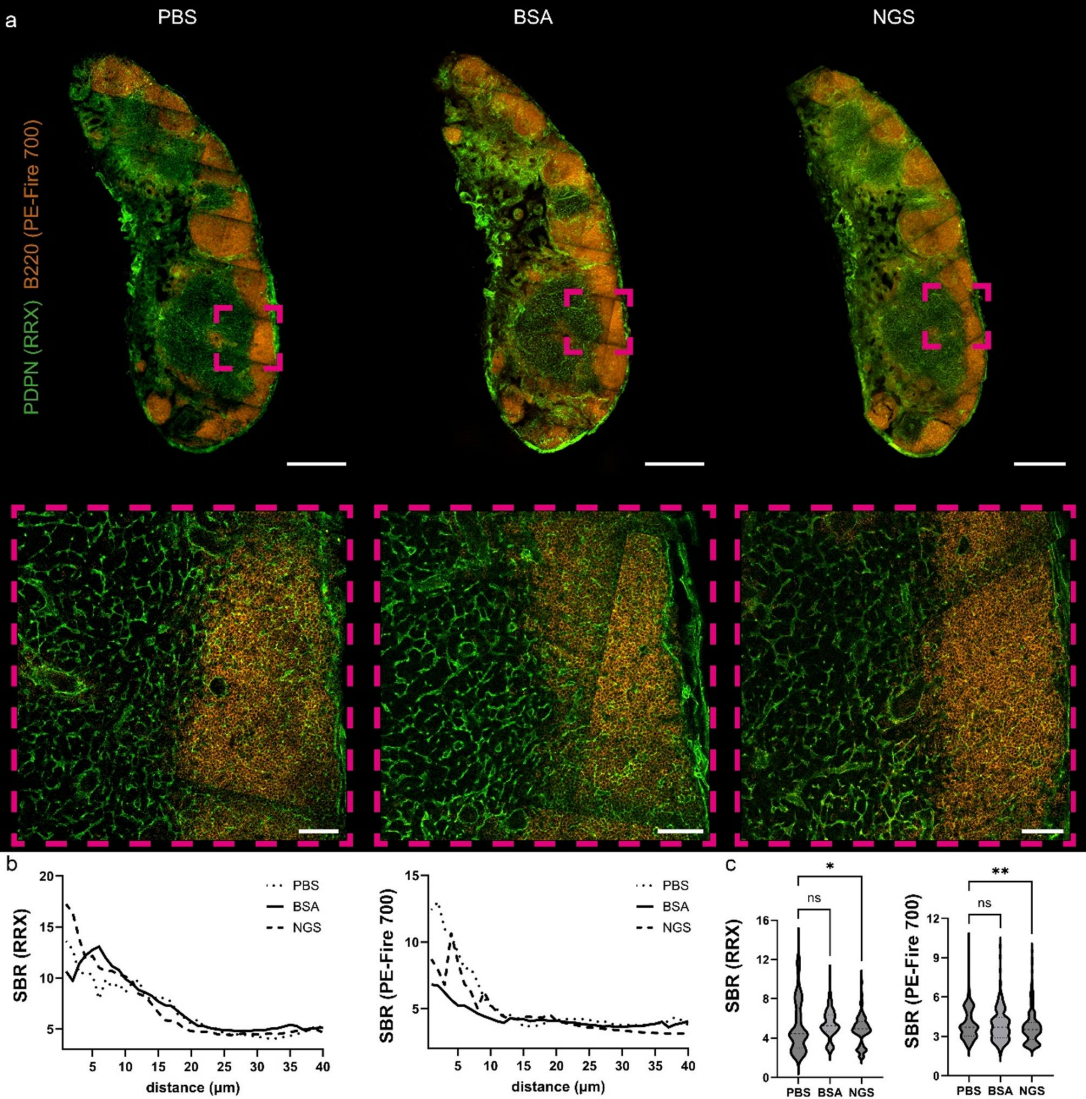
Immunofluorescence staining of murine lymph node tissue samples with and without the protein blocking step. (**a**) immunolabeling of murine lymph node against B220 (marker of B-lymphocytes) with primary antibody conjugated with Pe-Fire 700 (orange) and against podoplanin with secondary antibody conjugated with rhodamine red X (RRX, green), ROI is zoomed in lower panels. (**b**) mean SBR values of used fluorophores across 40 μm imaging range, *n* = 13–14 per group and (**c**) SBR values pooled from 15 to 40 μm imaging range, one-way ANOVA with Dunn’s multiple comparison test, *n* = 13–14 per group; **P < 0.01, *P < 0.05. Scale bar, 500 μm and 50 μm in zoomed areas.

Given that the absence of noticeable background staining and optimal SBR values in PBS (no block) group could be attributed to the use of antibodies conjugated to Alexa Fluor dyes—known for their excellent imaging properties—we conducted an additional experiment using antibodies conjugated to the less frequently used rhodamine red X (RRX) and PE-Fire 700 fluorophores. Once again, no nonspecific staining in PBS group and no qualitatively discernible differences were observed between the blocked and unblocked groups (Fig. 3a). Quantitative analysis revealed a similar pattern in the SBR curve as a function of imaging depth (Fig. 3b). Cumulative analysis for RRX and Pe-Fire 700 indicated no significant differences in SBR values between 15 and 40 micrometers for both PBS and BSA conditions, while slightly lower SBR values were observed in samples blocked with NGS (Fig. 3c).

**Fig. 4:**
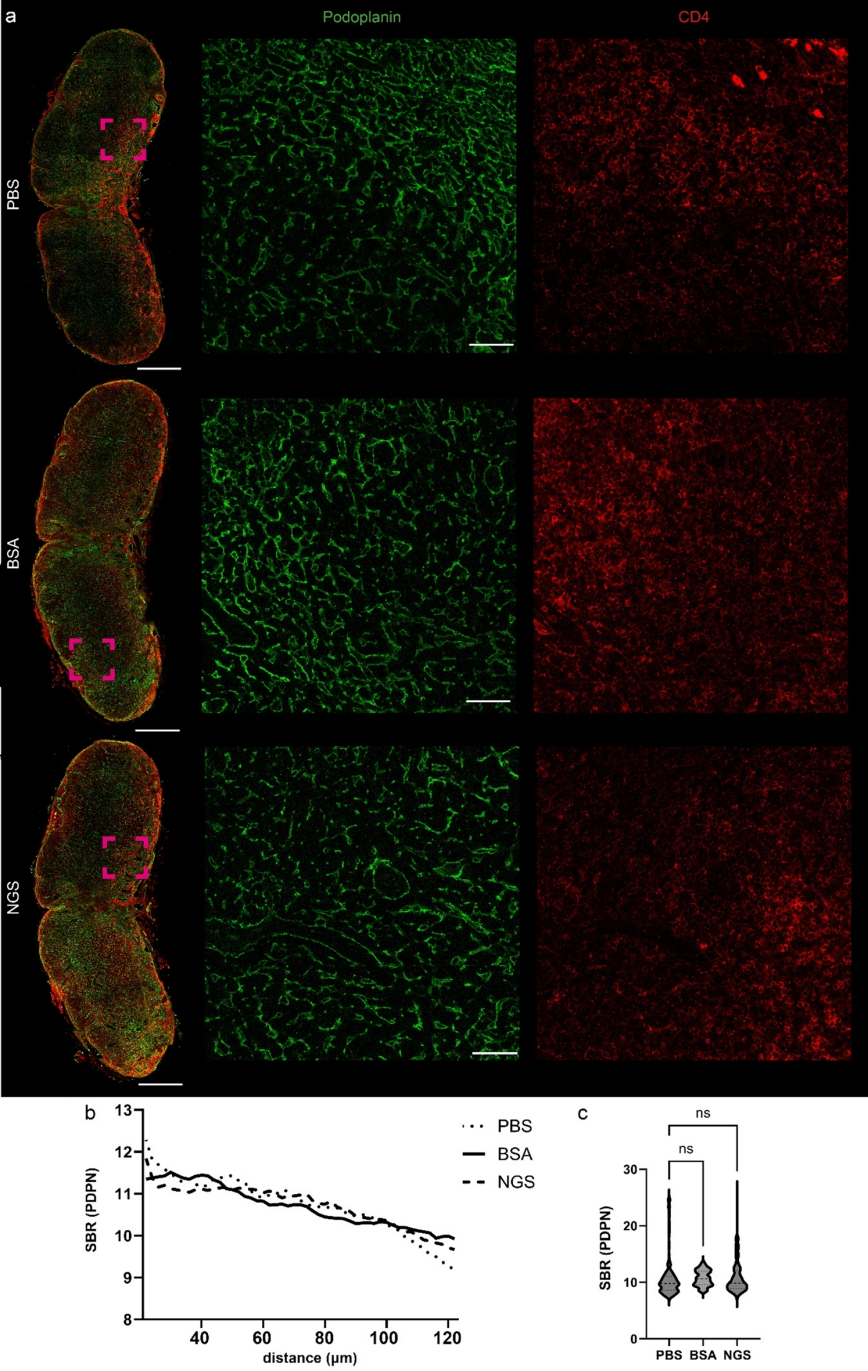
Immunofluorescence staining of thick murine lymph node tissue samples processed with and without the protein blocking step prior to incubation with primary Abs. (**a**) immunolabeling of murine lymph node against podoplanin (marker of fibroblastic reticular cells and lymphatic endothelial cells) and CD4 followed by secondary antibodies conjugated with AF-555 and AF-647, respectively. ROI is zoomed in lower panels. (**b**) mean SBR values of used fluorophores across 20–125 μm imaging range, *n* = 11–12 per group and (**c**) SBR values pooled from 20 to 120 μm imaging range, one-way ANOVA with Dunn’s multiple comparison test, *n* = 11–12 per group. BSA, bovine serum albumin, NGS, normal goat serum. Scale bar, 400 μm and 50 μm in zoomed areas.

Assuming that a larger volume of tissue increases the likelihood of nonspecific antibody binding, it was possible that the lack of blocking step might result in nonspecific signal in thick tissue sections. To test this, 125-µm mouse lymph node sections were stained for distinct cell populations – against podoplanin to visualize fibroblastic reticular cells (forming the lymph node stroma) and lymphatic endothelial cells and against CD4-positive lymphocytes - and cleared using the Ce3D TOC method, which is optimized for lymphoid tissues^18^. Again, no differences in specific or nonspecific signal were observed between the PBS (no block) and experimental groups (Fig. 4a). Quantitative assessment was limited to PDPN (as CD4-positive signal was too variable across 125-µm imaging depth) and revealed no difference between SBR values (Fig. 4b-c). Notably, the average SBR value for podoplanin within the 20–120 μm range was approximately 10, which was about twice as high as the value observed in the 15–40 μm range without TOC, further highlighting the added value of applying TOC methods - in this case, Ce3D.

**Fig. 5:**
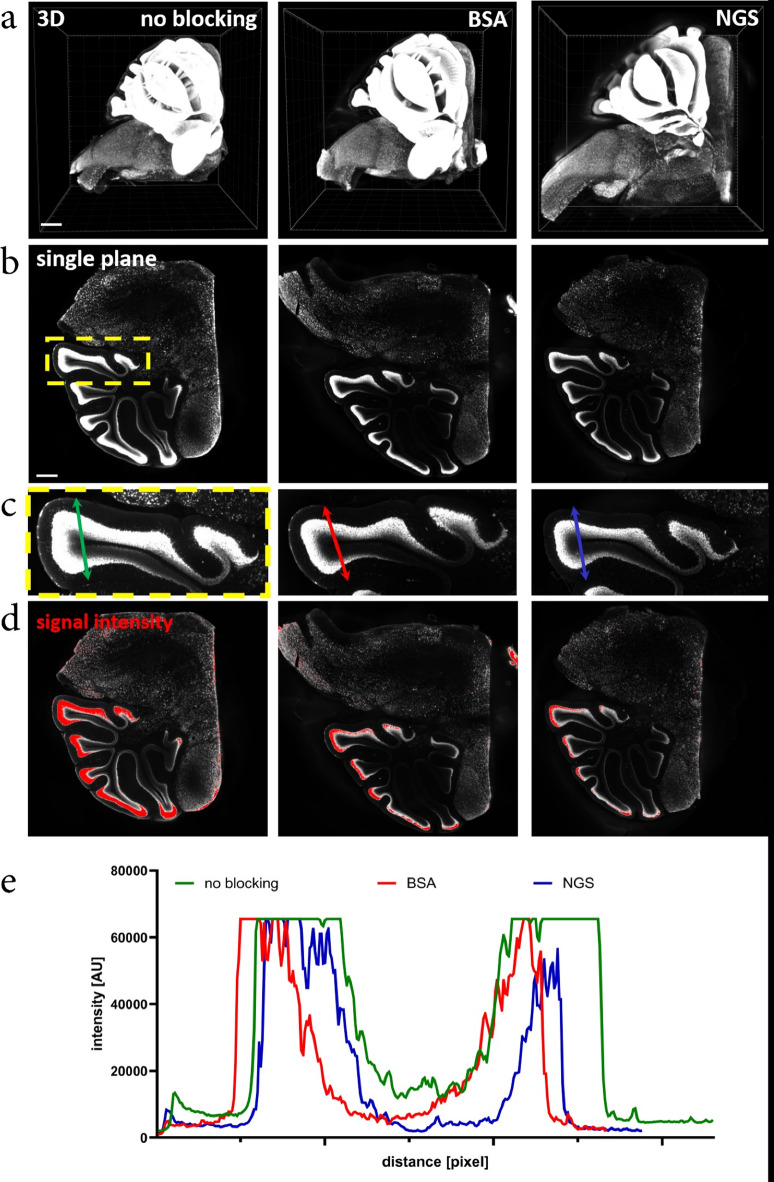
NeuN in cerebellum imaged using light-sheet microscope. (**a**) 3D reconstruction of images acquired using light-sheet microscope. (**b**) single plane taken from the middle of 3D Z-stack. Yellow ROI indicates zoom in (c). (**c**) Zoomed fragment of the cerebellum. Colored arrows indicate axis selected for NeuN signal intensity analysis (**d**). Red color indicates signal saturation. Note higher NeuN signal intensity when no blocking was used. (**e**) Signal intensity of NeuN antibody penetration. Scale bar in (a) 800 μm, (b) 400 μm.

Encouraged by these findings, we extended the study to large specimens of brain tissue. We stained cerebellar hemispheres without blocking or with BSA or NGS as a blocking agent. Tissues were optically cleared using the widely adopted iDISCO method and stained against structurally widespread antigen – NeuN, to check for both specificity of staining and possible qualitative difference in its distribution (Fig. 5). Remarkably, omitting the blocking step not only failed to introduce nonspecific signal, but also significantly improved the signal intensity and penetration of the specific signal.

**Fig. 6:**
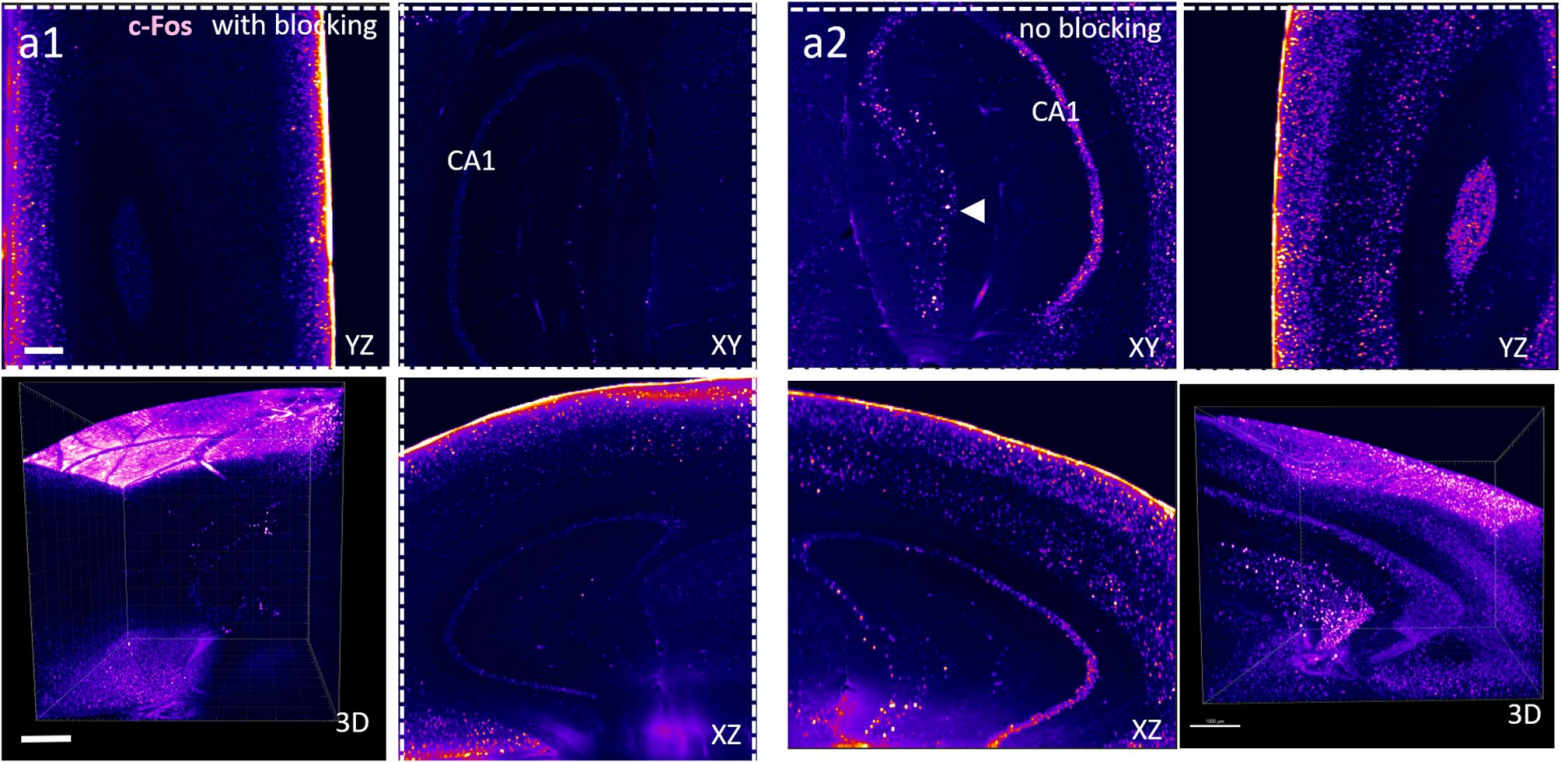
No blocking enhances antibody penetration into deeper layers in immunofluorescence staining combined with iDISCO clearing technique. a1-a2 iDISCO clearing combined with immunostaining for c-Fos after behavioral appetitive training in mouse brain imaged using light-sheet microscope. a1 – single planes in XYZ, standard iDISCO (with blocking), a2 single planes in XYZ – iDISCO performed without blocking step. Note increased penetration of antibody and signal with no blocking. c-Fos positive cell is marked with an arrowhead. The 3D panel depicts a reconstruction of the Z-stack covering a piece of cortex and the hippocampus. Scale bar in a1-a2 500 μm, in 3D panel 1000 μm.

Finally, for the exact comparison, we used two halves of the mouse brains, either with or without BSA blocking (selected as a more commonly utilized blocking agent) and used primary and secondary antibodies staining to target c-Fos. Imaging of PBS (no blocking) group performed after iDISCO clearing again revealed similar pattern of higher signal intensity (without introduction of non-specific antibody binding, Fig. 6).

## Discussion

In various fields, including science, there exist widespread practices and protocols that, despite advancements in technology and reagent development, remain unchallenged for years. A notable example is the practice of protecting stained samples from light exposure, even though the photobleaching of modern fluorophores requires very specific conditions—namely, the use of specialized reagents and intense light sources, as seen in methods like IBEX^19^ which enable iterative fluorophore bleaching and restaining with the same fluorophores (and, naturally, different primary antibodies). The findings presented by Buchwalow^17^ and subsequently corroborated by Jenvey^20^ suggest that a similar, currently unjustified assumption persists concerning the necessity of protein blocking during antibody-based staining protocols. While blocking may have minimal impact in the case of thin tissue sections stained briefly (typically up to overnight) and in small volumes, the situation is markedly different for thick, optically cleared samples, such as entire rodent brains, which require significantly more time and reagents. For instance, applying the iDISCO protocol to a mouse brain can take up to two weeks, with two days dedicated solely to the blocking step^21^. Given that BSA is commonly used not only during blocking but also in antibody staining steps, omitting BSA could potentially accelerate protocols and reduce costs by few up to dozen percent (the exact values will depend on the specific protocols employed in individual laboratories).

Building on the observations of Buchwalow, we sought to determine whether omitting the blocking step from immunofluorescence protocols, particularly for thick and optically cleared tissues, would negatively affect imaging quality. Interestingly, we found that the use of BSA as a blocking agent led to a reduced signal-to-background ratio (SBR) for each of three commonly used fluorophores (AF488, AF555, and AF647), under identical imaging conditions. This effect was not uniform across all blocking methods, as SBR for NGS-blocked samples was statistically lower only for AF488, with no significant differences observed for AF555 and AF647 compared to unblocked controls. Conversely, in case of RRX and Pe-Fire 700 fluorophores, cumulative SBR was slightly lower with NGS versus PBS and BSA. However, under no experimental conditions did we observe that the omission of the blocking step resulted in nonspecific signals or compromised signal quality. This finding persisted even when using lymphoid organs such as lymph nodes and thymus, which are known to have the highest density of Fc receptors on average, imputed to facilitate the capture of antibody Fc fragments.

It is important to note that the challenge of proper antibody penetration in thick, optically cleared samples is well-documented and has been addressed through various approaches^22^. For instance, Kim et al.^23^ developed stochastic electrotransport, Murray et al.^14^ and recently Yau et al.^15^ introduced reagents that transiently reduce antibody binding affinity to antigens (allowing sufficient penetration time before removal of the reagent), and Susaki et al.^16^ observed that fixed animal tissue behaves like a hydrogel, antibody binding properties of which can be optimized by adjusting parameters such as temperature, ionic strength, detergent concentration, and limited tissue digestion. Due to their size, antibodies exhibit slow penetration into fixed tissues, achieving a depth of 100–1000 μm per day^22^. Therefore, whenever feasible, the use of smaller epitope-binding fragments is recommended instead of whole antibodies^24^. This challenge is exacerbated in cases where the organ exhibits a high density of a given epitope, creating a dense network of antibodies that further hinder deep tissue penetration. In our experiments, using mouse brain hemispheres and staining against c-Fos (using primary and secondary antibodies), we observed that the application of BSA resulted in a substantial accumulation of signal on the tissue surface (Fig. 6a1). Interestingly, this effect was not observed in the contralateral brain hemisphere, where BSA was not applied. Similarly, omission of blocking step in case of mouse cerebellum stained against NeuN resulted in both higher penetration of antibodies and signal intensity. Although the precise mechanism underlying this phenomenon requires further investigation, our observations suggest that blocking agents like BSA and NGS either interacts with the tissue surface or deposits within it in a manner that impedes antibody penetration into the organ’s deeper regions.

In aggregate, our findings derived from 50 to 125 μm tissue slices and mouse brain hemispheres and cerebella suggest that another, perhaps counterintuitive, strategy for improving signal detection is the simple omission of the blocking step - at least with commonly used agents like BSA and NGS. Although it remains uncertain whether these effects are universal across all tissue types and antibodies, based on our observations and given the potential substantial time and cost savings, preliminary experiments to validate this approach should be considered before initiating large-scale studies.

## Methods

### Mice

Tissues were obtained from inbred C57BL/6J mice (bred at the Center for Experimental Medicine in Białystok and the M. Mossakowski Medical Research Center in Warsaw or the Nencki Institute of Experimental Biology). The animals were provided a controlled environment (temperature 24 °C, 12/12 light/dark cycle) with *ad libitum* access to water and feed. The animals were euthanized by cervical dislocation following exposure to isoflurane (FDG9623, Baxter). Isolated tissues were fixed for 24 h in a 4% paraformaldehyde solution (BD Cytofix/Cytoperm™, No. 554722, BD Biosciences) at 4 °C before being used in experiments. All procedures were conducted in accordance with the Directive of the European Parliament and Council (2010/63/EU) and Polish Law. Mice were solely tissue donors, meaning no ethical committee approval was required.

### Preparation of tissue sections

The fixed organs were embedded in a 3% w/v agarose solution (Sigma-Aldrich) in water and subsequently sectioned to the desired thickness using a Vibratome (VT1000S, Leica). The sections were stored in 1× phosphate-buffered saline (PBS, Sigma-Aldrich) supplemented with 0.05% sodium azide (NaN3, Sigma–Aldrich).

### Immunofluorescence staining

Thin tissue sections (50–125 μm) were incubated in a 0.3% Triton X-100 (Sigma-Aldrich) in PBS or blocking solution consisting of either 3% BSA, NGS or Fc block (1:100, BD Pharmingen™ Purified Rat Anti-Mouse CD16/CD32) in 0.3% Triton X-100 (Sigma-Aldrich) in PBS at room temperature. The sections in the blocking solution were exposed to a mixture of primary antibodies or directly conjugated with fluorochromes (the list and concentrations of antibodies used are presented in Supplementary Table 1). The following day, the sections were washed in 0.3% Triton X-100 in PBS (3 × 10 min). For primary antibodies, after the final wash, secondary antibodies conjugated with fluorophores were added and incubated overnight. Sections were then rinsed in PBS (3 × 10 min) and mounted on slides. All staining and washing steps were performed on a Mini–Shaker PSU-2T (BS-010155-AAG, Biosan). Fluoromount-G (Thermo Fisher Scientific) was used for mounting 50-µm thick samples, while the 125-µm thick samples were mounted in the C_e_3D solution, as detailed below.

### C_e_3D

The solution was prepared according to a published protocol^18^. In brief, a 40% v/v solution of N-methylacetamide (M26305-100G, Sigma–Aldrich) in PBS was prepared and used to dissolve Histodenz (D2158-100G, Sigma–Aldrich) to 86% w/v at 37 °C. After complete dissolution, achieved in 5–6 h, Triton X-100 (0.1% v/v, Sigma–Aldrich) and 1-thioglycerol (0.5% v/v, M1753-100ML, Sigma–Aldrich) were added to the solution. Incubation was carried out for 24 h with gentle rotation.

### Confocal imaging

For imaging, a Leica TCS SP8 confocal microscope (Leica Microsystems) equipped with a Navigator module and HC PL APO CS2 40×/1.30 oil immersion objective was employed. Excitation was achieved using a white light laser (WLL) or argon laser at appropriate wavelengths for specific fluorophores. Detection was predominantly performed using hybrid detectors, HyD, at a resolution of 1024 × 1024, with a speed of 400–600 Hz. Optical microscopy experiments were performed at the Laboratory of Imaging Tissue Structure and Function which serves as an imaging core facility at the Nencki Institute of Experimental Biology.

### Signal-to-background ratio

Data was analyzed similarly to Nürnberg et al.^25^. Briefly, z-stacks were acquired and analyzed using ImageJ. First, image was duplicated and a median filter (radius 1) was applied and a threshold range was manually set in upper regions of the sample, to cover areas defined as background, and converted into binary masks. Finally, for determination of signal intensity, an automated thresholding mechanism (IsoData) was applied independently to each optical section and images were converted into binary masks, which were used to measure signal intensities from initial stacks. SBR was calculated for each optical section separately.

### iDISCO protocol and light-sheet fluorescence microscopy

Two hours after access to reward (5% sucrose solution) mice were injected with sodium pentobarbital and perfused using 0.01 M PBS (pH 7.4)/heparin (5 IU/ml final concentration) and 4% PFA/0.01 M PBS. Brains were isolated and postfixed in 4% PFA for 12 h. Optical tissue clearing was performed on two hemispheres separately using iDisco +^26,27^ with immunostaining for c-Fos and NeuN. Brains were cut sagittally into two equal parts. NeuN staining was performed on cerebella only. Briefly, initial dehydration was performed in graded methanol/water solutions (20%, 40%, 60%, 80%, 100%, and 100%) for six cycles of 1 h each at room temperature with rotation. This was followed by overnight dehydration in a 2:1 dichloromethane (DCM): methanol solution under similar conditions. Samples were then washed twice with 100% methanol for 30 min each, bleached overnight in a 1:5 mixture of 5% hydrogen peroxide in methanol at 4 °C on a rotor, and rehydrated in reverse methanol/water gradients (60%, 40%, 20%) followed by phosphate-buffered saline (PBS) over four 1-hour cycles at room temperature with rotation. Subsequent washes were performed twice with PTx.2 (1-hour each) at room temperature with rotation. Next samples were permeabilized (PTx.2, glycine, DMSO) for 2 days at 37 °C with rotation and incubated in blocking solution (PTx.2, 6% BSA/PTwH, DMSO). Immunostaining was conducted with primary antibodies for c-Fos (Synaptic Systems) diluted in PTwH/DMSO/3% BSA for 7 days at 37 °C with rotation. Next samples were washed in PTwH (five cycles: four 30-minute washes and one overnight) at 37 °C. Secondary antibody (anti-guinea pig-AlexaFluor 647) staining was performed using PTwH/3% BSA for 7 days at 37 °C, followed by similar PTwH washes. Final dehydration included graded methanol/water steps (20%, 40%, 60%, 80%, and 100%) over five 1-hour cycles, overnight incubation in 100% methanol, and subsequent steps in 2:1 DCM/methanol for 3 h on a shaker, followed by two 15-minute washes in 100% DCM. Clearing was achieved with dibenzyl ether (DBE) overnight at room temperature. For “no blocking” variant, blocking step was omitted. BSA was also not added to immunostaining cocktail. For NeuN staining on cerebella we used 3%BSA or 10% NGS (normal goat serum) as a blocking reagents, aNeuN Rb antibody (1:500 MABN140, Merck) and aRb AlexaFluor647 antibody (1:500, A-21244, ThermoFisher).

Cleared samples were imaged using light-sheet microscope (Ultramicroscope II, LaVision BioTec GmbH, Bielefeld, Germany) equipped with 4× objective NA 0.5, WD 6 mm, and Hamamatsu Orca-Flash4.0 camera (Spectra Services, Ontario, NY, USA). The 638 nm laser was used for excitation of fluorophores. Data were collected with 5-µm Z step and resolution in XY 0.89 × 0.89 μm. As the specimen was larger than the field of view of the microscope, imaging was performed as a series of 3D tiles that were later stitched, fused, and exported as a single tiff stack using TeraStitcher v 1.10.4. 3D images were analyzed using Imaris (Bitplane Inc., Belfast, UK). For cerebella and NeuN imaging we did not perform mosaic acquisition as it was not necessary. Images were collected with 5-µm Z step and resolution in XY 2.46 × 2.46 μm. Pixel intensity profiles were analyzed in Zen Software (Zeiss).

### Statistical analysis

Data were considered statistically significant for *p* < 0.05. The number of analyzed elements is provided in the figure legends. Statistical analyses were performed using GraphPad Prism 10 (GraphPad Software). One-way analysis of variance (ANOVA) with Dunn’s multiple comparison test was applied for non-parametric data.

## Electronic supplementary material

Below is the link to the electronic supplementary material.

Supplementary Material 1

## Data Availability

All raw data supporting the findings of this study are available from corresponding author upon request.
