# Supplementary material for: Use of bovine serum albumin might impair immunofluorescence signal in thick tissue samples
Source: Sci Rep. 2025 Jul 1;15:21920. doi: 10.1038/s41598-025-06876-z (PMC12215187; doi:10.1038/s41598-025-06876-z)
Supplement: Supplementary file 1 — Supplementary Material 1 [file 41598_2025_6876_MOESM1_ESM.docx]

**Supplementary Information**

**Use of Bovine Serum Albumin Might Impair Immunofluorescence Signal in Thick Tissue Samples**

**Chwastowicz et al.**

**Supplementary Figures 1 - 2**


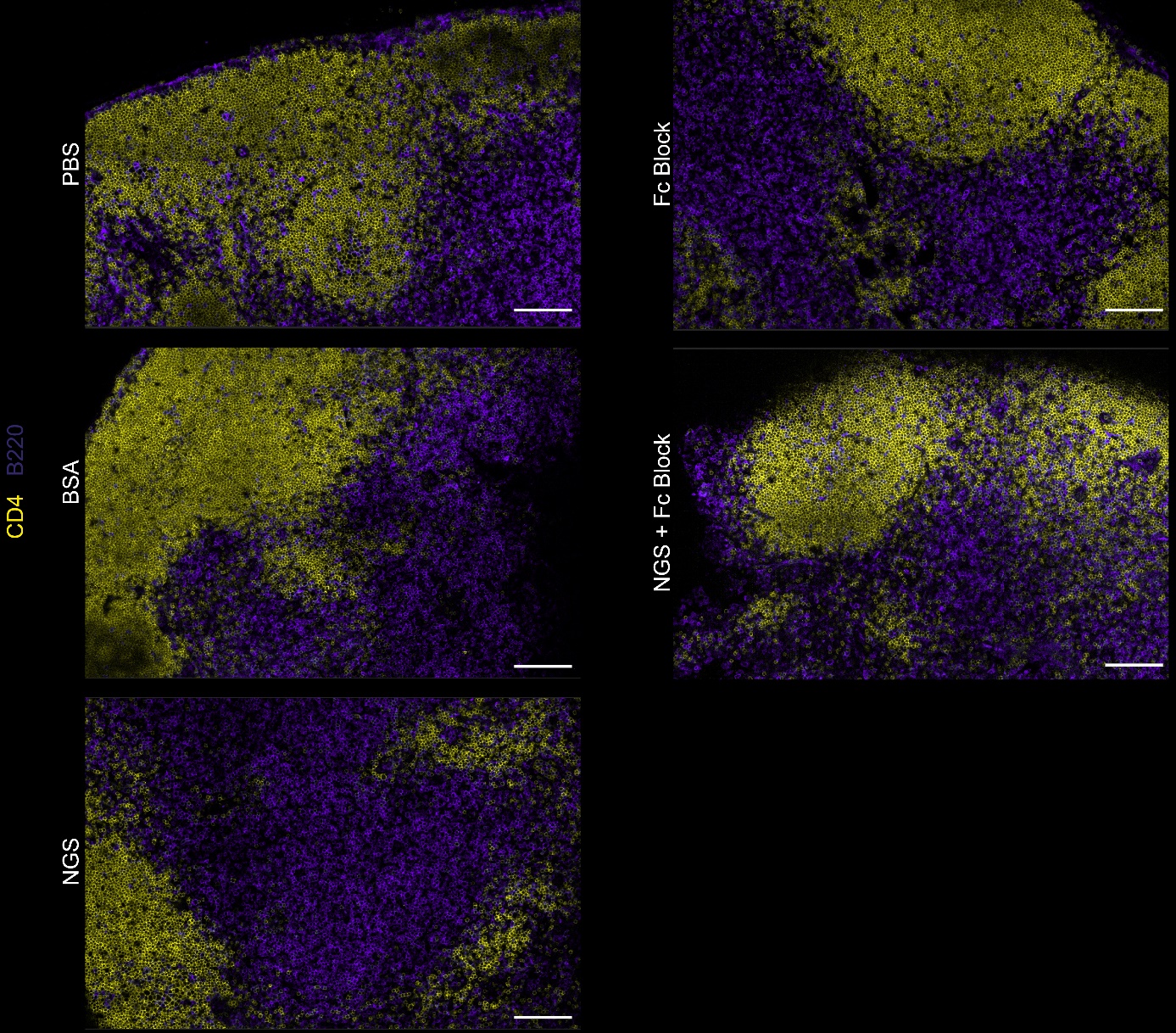


**Supplementary Figure 1: Enlarged images presented in Figure 1**

Immunolabeling of murine lymph node against B220 (marker of B-lymphocytes) and CD4 (marker of T-helper lymphocytes) with secondary antibody conjugated with AF-488 and AF-647, respectively.

BSA, bovine serum albumin, NGS, normal goat serum. Scale bar, 100 µm.


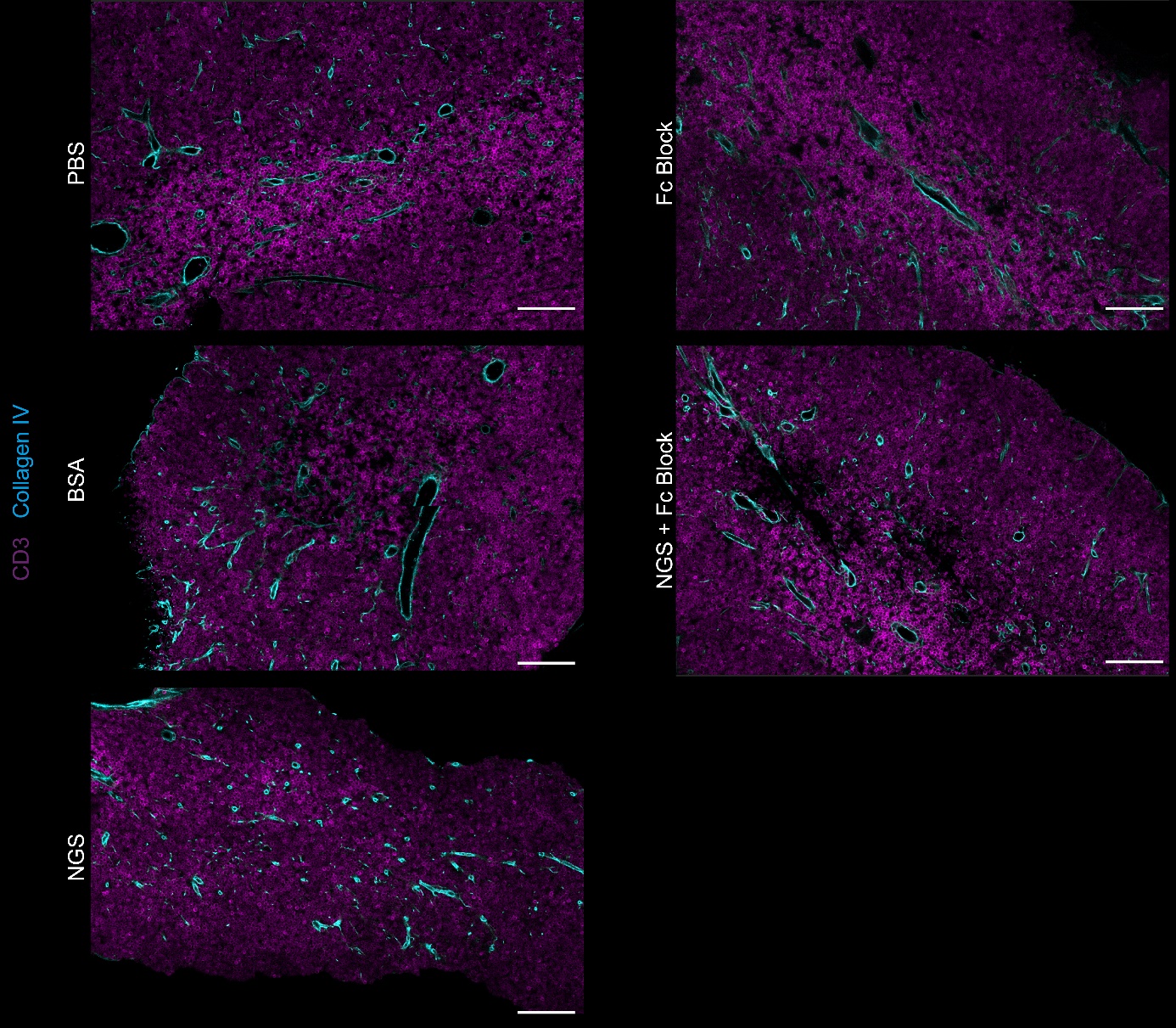


**Supplementary Figure 2: Enlarged images presented in Figure 1**

Immunolabeling of murine thymus against collagen IV with secondary antibody conjugated with AF-568 and against CD3 (marker of T lymphocytes) with AF-647 directly conjugated antibody.

BSA, bovine serum albumin, NGS, normal goat serum. Scale bar, 100 µm.
